# Impact of Methotrexate and 7‐Hydroxymethotrexate Exposure on Renal Toxicity in Pediatric Non‐Hodgkin Lymphoma

**DOI:** 10.1002/cam4.70516

**Published:** 2025-01-16

**Authors:** Hao Bing, Yi Ma, Jiamin Xu, Qixian Ling, Yanlong Duan, Libo Zhao

**Affiliations:** ^1^ Clinical Research Center Beijing Children's Hospital, Capital Medical University Beijing China; ^2^ Department of Pharmacy Peking University Third Hospital Beijing China; ^3^ Department of Oncology Beijing Children's Hospital, Capital Medical University Beijing China

**Keywords:** methotrexate, nephrotoxicity, peak concentration, population pharmacokinetics

## Abstract

**Background:**

7‐Hydroxymethotrexate (7‐OHMTX) is the main metabolite in plasma following high‐dose MTX (HD‐MTX), which may result in activity and toxicity of the MTX. Moreover, 7‐OHMTX could produce crystalline‐like deposits within the renal tubules under acidic conditions or induce renal inflammation, oxidative stress, and cell apoptosis through various signaling pathways, ultimately leading to kidney damage. The objectives of this study were thus to explore the exposure–safety relationship of two compounds and search the most reliable marker for predicting HDMTX nephrotoxicity.

**Method:**

A total of 280 plasma concentration data (140 for MTX and 140 for 7‐OHMTX) for 60 pediatric patients with non‐Hodgkin lymphoma (NHL) were prospectively collected. Plasma MTX and 7‐OHMTX concentrations were determined using a high‐performance liquid chromatography tandem mass spectrometry (HPLC–MS/MS) method. A nonlinear mixed effect model approach was used to build a joint population pharmacokinetic (PopPK) model. After validation, the model estimated the peak concentration (*C*_max_) and area under the curve within the initial 48 h (AUC_0‐48h_) of the patients after drug administration by Bayesian feedback. The receiver operating characteristic (ROC) curves were generated to identify an exposure threshold associated with nephrotoxicity.

**Results:**

A three‐compartment chain model (central and peripheral compartments for MTX and central compartment 7‐OHMTX) with the first‐order elimination adequately characterized the in vivo process of MTX and 7‐OHMTX. The covariate analysis identified that the aspartate aminotransferase (AST) was strongly associated with the peripheral volume of distribution of MTX. Moreover, the *C*_max_ of MTX and 7‐OHMTX showed significant differences (*p* < 0.0001, *p* = 0.0472, respectively) among patients with or without nephrotoxicity. Similarly, individuals with nephrotoxicity also exhibited substantially higher ratio of 7‐OHMTX to MTX peak concentration and the sum of MTX + 2.25 times the concentration of 7‐OHMTX (*p* < 0.0001, *p* = 0.0426, respectively). By ROC analysis, the *C*_max_ of MTX and 7‐OHMTX had the greatest area under the curve (AUC) values (0.769 and 0.771, respectively). A *C*_max_ threshold of 9.26 μmol/L for MTX or a *C*_max_ threshold of 0.66 μmol/L for 7‐OHMTX was associated with the best sensitivity/specificity for toxicity events (MTX: sensitivity = 0.886; specificity = 0.70; 7‐OHMTX: sensitivity = 0.886; specificity = 0.70).

**Conclusions:**

We demonstrated that the *C*_max_ of MTX and 7‐OHMTX were the most reliable markers associated with nephrotoxicity and proposed a *C*_max_ threshold of 9.26 μmol/L for MTX and 0.66 μmol/L for 7‐OHMTX as the point with a high risk of nephrotoxicity. Altogether, this study may contribute to crucial insights for ensuring the safe administration of drugs in pediatric clinical practice.

**Abbreviations**
7‐OHMTX: 7‐hydroxymethotrexate
AIC: Akaike information criteria
AKI: acute kidney injury
ALP: alkaline phosphatase
ALT: alanine aminotransferase
AST: aspartate aminotransferase
AUC_0‐48h_
: area under the concentration–time curve within the initial 48 h
BMI: body mass index
BSA: body surface area
CI: confidence interval
CL: central clearance
*C*_max_
: peak concentration
CObs: observed concentrations
CWRES: conditional weighted residuals
Fm: fraction of metabolism
GFR: glomerular filtration rate
GOF: goodness of fit
HD‐MTX: high‐dose methotrexate
HPLC–MS/MS: high‐performance liquid chromatography tandem mass spectrometry
IPRED: individual predicted concentrations
IQR: interquartile range
LD50: lethal dose 50
LLOQ: lower limit of quantitation
NHL: non‐Hodgkin lymphoma
OFV: objective function value
pcVPC: prediction‐corrected visual prediction checks
PopPK: population pharmacokinetics
PRED: population predicted concentrations
*Q*
: intercompartmental clearance
ROC: receiver operating characteristic
RSE%: percentage relative standard error
SCr: serum creatinine
SD: standard deviation
TAD: time after dose
TB: total bilirubin
TBA: total bile acid
TP: total protein
tv: the population typical value of the parameter
UA: uric acid
*V*
: volume of distribution
V2: volume of the second compartment
γ‐GGT: gamma‐glutamyl transpeptidase
*η*
: interindividual variability of parameters
*θ*_AST_
: effect of AST on Vp_MTX_
*σ*
: residual variability

## 1 Introduction

Non‐Hodgkin lymphoma (NHL) are malignant disorders that originate from cells of the immune system, including B, T, and natural killer (NK) cells, and manifest predominantly as lymphadenopathy or solid tumors [1]. The most recent World Health Organization classification of NHL has more than 50 distinct subtypes, making it a complicated and dynamic categorization [2]. In some circumstances, more than half of patients can be cured with current medication; however, many subtypes of the disease are incurable with current management tactics [3].

Methotrexate (MTX), an aminopterin analog, is a folate antagonist that inhibits the de novo synthesis of purines and pyrimidines by inhibiting thymidylate synthase and dihydrofolate reductase, resulting in antimalignant, anti‐inflammatory, and immunosuppressive properties [4, 5]. High‐dose MTX (HD‐MTX) is recommended as an important component of chemotherapy for NHL, which can significantly improve the survival of patients [6, 7, 8], but it also brings some life‐threatening toxicity, such as mucositis [9], hepatotoxicity [10], nephrotoxicity [11], and myelosuppression [12]. 7‐Hydroxymethotrexate (7‐OHMTX) formed in the liver is the main metabolite in plasma following HD‐MTX [5], which may result in activity [13] and toxicity [14] of the drug, and also affects the pharmacokinetics and pharmacodynamics of MTX [15]. With intravenous administration, approximately 80% to 90% of MTX is excreted in the urine and 7‐OHMTX is likewise cleared by the kidneys but more slowly than MTX [16, 17]. Moreover, 7‐OHMTX has 3–5 folds lower water solubility than MTX over the pH range 5.0–7.0, which could produce crystalline‐like deposits within the renal tubules and subsequently lead to kidney damage [15, 18]. Moreover, a previous study has also indicated that 7‐OHMTX is the main cause of nephrotoxicity. It induces endoplasmic reticulum stress through excessive accumulation in the kidneys, leading to cell apoptosis via the PERK/CHOP signaling pathway [19]. In addition, MTX itself can also cause kidney damage through inflammatory response, oxidative stress, apoptosis, and other pathways [20, 21, 22]. Adequate hydration and alkalinization prior to administration of HD‐MTX maintain the urine pH above 7.0, which can reduce the incidence of these adverse reactions [23]. Currently, numerous methods have been developed for regular monitoring of MTX and 7‐OHMTX concentrations, which guide the dose of leucovorin and strategy [24, 25, 26]. However, due to the large interindividual variances in the pharmacokinetic course of HD‐MTX, some patients may also have severe adverse reactions after supportive treatment, especially renal injury. Renal damage causes decreased MTX clearance and prolonged exposure to toxic concentrations, worsening renal function and exacerbating nonrenal side effects [11]. Previous population pharmacokinetic (PopPK) studies have shown no significant associations between the two pharmacokinetic variables (model‐derived 7‐OHMTX AUC_0‐∞_ and the sum of model‐derived MTX + 7‐OHMTX AUC_0‐∞_) and the toxicity events observed in infants and young children with brain tumor population [27]. Additionally, an in vitro study also revealed that 7‐OHMTX had higher renal cytotoxicity than MTX, as evidenced by a lower lethal dose 50 (LD50) [19]. However, the current PopPK studies of HD‐MTX rarely incorporate 7‐OHMTX concentrations into the model, which is inconvenient to predict metabolite exposure. Currently, to the best of our knowledge, there is a lack of research on the relationship between drug exposure and nephrotoxicity, and no information is available regarding the specific cutoff value for nephrotoxicity following therapy.

Herein, the objectives of the present study were to [1] establish a PopPK model describing the pharmacokinetics of MTX and 7‐OHMTX in pediatric patients with NHL and evaluate the exposure–safety relationship of two compounds, and [2] explore the most reliable markers for predicting HDMTX nephrotoxicity in order to provide guidance for enhancing drug safety.

## 2 Methods

### 2.1 Study Population

Pediatric patients with NHL receiving intravenous HDMTX either as single agent or in combination with other cytotoxic drugs treated at the Beijing Children's Hospital from September 2022 to July 2023 were included in the analysis. The inclusion criteria were as follows: (1) age less than 18 years, (2) clinically diagnosed NHL according to guidance [28], and (3) treated with HD‐MTX. This study was in compliance with the Declaration of Helsinki and approved by the Beijing Children's Hospital Medical Science Research Ethics Committee (No. [2022]‐E‐118‐Y). Patients provided written informed consent prior to enrollment.

Patients were excluded if they were allergic to MTX, the clinical diagnosis was unclear or the information was incomplete, they did not undergo blood sample collection according to the prescribed time, and they participated in other clinical trials prior to screening.

### 2.2 Dose Regimen, Sampling, and Assay of Plasma MTX

MTX was given in different doses depending on the pathological classification, tolerance, and genotypes for transporters and metabolic enzymes of patients. In general, the dose is 3 g/m^2^ for B‐cell lymphoma and 5 g/m^2^ for T‐cell lymphoma.A 24‐h intravenous regimen was employed for lymphoblastic lymphoma, with a fast infusion of 1/10 of the entire dose within the initial 30 min, followed by a continuous infusion of the remaining dose over a duration of 23.5 h. For the other pathological types, a fast infusion regimen within 3–4 h is usually used.

Prior to administration, the patient was given adequate hydration and urine alkalinization. Leucovorin was administered 42 h after the start of treatment, with subsequent doses given every 6 h. Simultaneously, blood samples were collected from 45 h after MTX administration to monitor the blood concentration of MTX every 24 h until it dropped below 0.1 μmol/L.

The plasma samples were prepared by centrifugation at 3000 rpm for 5 min and were subjected to protein precipitation. The plasma concentration of MTX and 7‐OHMTX was determined by a validated high‐performance liquid chromatography tandem mass spectrometry (HPLC–MS/MS) method established in our laboratory with a lower limit of quantitation (LLOQ) of 1 ng/mL.

### 2.3 PopPK Model Building

#### 2.3.1 Base Structural Model

Descriptive statistical analysis of the data was implemented using SPSS 26 (IBM Ltd., USA). Prisim Graphpad (version 9.4.1, GraphPad Software, USA) was used for data manipulation and visualization. PopPK models were developed in Phoenix 8.3 (Certara USA Inc., Princeton, NJ, USA) using a nonlinear mixed effects model (NLME). A sequential two‐step analysis approach to modeling building was implemented [29]. In general, the modeling process was performed as follows: (1) development of a base structural and residual model that best describes the data; (2) screening and inclusion of covariates; and (3) evaluation and validation of the model.

A PopPK model for MTX was developed, followed by the establishment of a PopPK model for 7‐OHMTX with the pharmacokinetic parameters of the MTX being held constant. One‐, two‐, or three‐compartment distributions with first‐order elimination kinetic models were used to evaluate MTX and 7‐OHMTX as base structural models, respectively. The one‐compartment model parameters included the volume of distribution (*V*) and central clearance (CL). The two‐compartment structural model, intercompartmental clearance (*Q*), and volume of the second compartment (V2) were included. To prevent problem of calculating an independent parameter, the fraction of metabolism (Fm) of MTX that was metabolized to 7‐OHMTX was fixed at 10% in accordance with previous study [30]. A first‐order conditional estimation method with the extended least squares method (FOCE ELS) was used to estimate population typical parameter values and their interindividual variability. The interindividual variability was described by the following multiplicative exponential structure equation and was assumed to be normally distributed (Equation 1):

(1) $$\theta_{i}=\theta_{\mathrm{tv}}∙e^{\eta_{i}}$$

where *θ*_*i*_ represents the individual parameter value in the ith patient, *θ*_tv_ is the typical value of population parameters, and *η*_*i*_ describes the variability of the parameter between the individual and population with a mean of 0 and a variance of *ω* ^2^.

In addition, residual error was described by additive, proportional, and combined additive and proportional residual models, where the equations are as follows (Equations (2), (3), (4)):

(2) $${\text{CObs}}_{\mathrm{ij}}={\text{CPred}}_{\mathrm{ij}}+\varepsilon_{\mathrm{ij}1}$$

(3) $${\text{CObs}}_{\mathrm{ij}}={\text{CPred}}_{\mathrm{ij}}\times \left(1+\varepsilon_{\mathrm{ij}2}\right)$$

(4) $${\text{CObs}}_{\mathrm{ij}}={\text{CPred}}_{\mathrm{ij}}\times \left(1+\varepsilon_{\mathrm{ij}2}\right)+\varepsilon_{\mathrm{ij}1}$$

where CObs_*ij*_ and CPred_*ij*_ represent the observed and predicted concentrations of the *i*th patient at the *j*th sampling point, respectively. *ε*_*ij*1_ is the additive residual and ε_ij2_ is the proportional residual, both of which are distributed with a mean of 0 and a variance of *σ* ^2^. Meanwhile, allometric scaling based on the bodyweight was applied to the PK parameters. An allometric power model with fixed power exponents of 0.75 for clearances and 1.0 for volumes of distribution was also tested [31]. The base structural model was selected according to the minimum objective function value (OFV, equal to −2 log‐likelihood), Akaike information criteria (AIC), the precision of parameter values (according to standard error and coefficients of variation of parameter estimates), and graphical analyses of the goodness of fit (GOF).

#### 2.3.2 Covariate Model Development

The impact of covariates on pharmacokinetic parameters was evaluated. Prior to covariate screening, a graphical analysis was conducted to assess the correlation between interindividual variance of pharmacokinetic parameters and covariates. The potential covariates were subsequently tested for (i) physiological and clinical significance and (ii) a reduction in the interindividual variability, including demographic information, pathological type, alanine aminotransferase (ALT), aspartate aminotransferase (AST), alkaline phosphatase (ALP), gamma‐glutamyl transpeptidase (γ‐GGT), total protein (TP), total bilirubin (TB), total bile acid (TBA), uric acid (UA), serum creatinine (SCr), and glomerular filtration rate (GFR) levels.

The covariate model was built using a stepwise modeling method. The stepwise procedure was divided into three steps, including one‐by‐one filtering, forward inclusion, and backward elimination. The covariates were individually examined, and only those that demonstrated significant improvements in the model performance were included in the multivariate analysis. Screened covariates were then assessed by forward addition and backward exclusion. In the forward inclusion process, a covariate was included if the −2LL value decreased by more than 3.84 (*p* < 0.05). In the backward exclusion process, a covariate was considered significant if the −2LL value increased by greater than 7.88 (*p* < 0.005) after removing the covariate from the model. Continuous variables were standardized at their median values, and the effects of each covariate on parameters were assessed using power functions (Equation 5):

(5) $$\theta_{i}=\theta_{\mathrm{tv}}∙{\left({\mathrm{cov}}_{i}/{\mathrm{cov}}_{\text{median}}\right)}^{\theta }$$

where cov_i_ and cov_median_ represent the *i*th individual and median value of the covariate, respectively, and *θ* stands for the estimated values of the covariate effect. Indicator variables were used to incorporate categorical covariates into the population model, and the effect of these covariates on each parameter was assessed using a scale function for example (Equation 6):

(6) $$\theta_{i}=\left\{\begin{array}{c} \theta_{\mathrm{tv}}\;\left(\text{if gender}=1\right)\\ \theta_{\mathrm{tv}}∙\left(1+\theta \right)\;\left(\text{if gender}=2\right) \end{array}\right.$$

where 1 or 2 denote male or female gender, respectively. In addition to statistical significance, a covariate was excluded from the model if the reduction in variability was < 5% [32].

### 2.4 Model Evaluation

To describe the fitting degree of final model, GOF plots including plots of observed concentrations (CObs) versus individual‐predicted and population‐predicted (IPRED and PRED) concentrations, plots of conditional weighted residuals (CWRES) over population‐predicted concentrations, and CWRES versus time after dose (TAD) were employed for model evaluation.

Prediction‐corrected visual prediction checks (pcVPC) and bootstrap methods were used to simultaneously assess the predictive performance and stability of the model. Bootstrapping was employed to create a fresh collection of samples by the process of randomly sampling and replacing data points from the original dataset. The process was iterated 1000 times to create 1000 simulated datasets from the identical population. Each dataset was then analyzed using the final model, resulting in a unique set of parameter estimates for each dataset. After resampling the datasets, the 2.5th and 97.5th percentiles, as well as the medians of the calculated parameters, were compared to the final model estimates. In addition, we generated simulated values by fitting the observed values 1000 times using pcVPC and determined the 2.5th, 50th, and 97.5th percentiles of the simulated and observed values. The visual congruence between the simulated and observed data was assessed.

### 2.5 Simulation and Statistical Analysis

On the basis of the established final PopPK model, the peak concentration (*C*_max_) of MTX and7‐OHMTX under several dosage regimens was predicted using Bayesian feedback. The C_max_ of the rapid infusion regimen refers to the concentration reached at the end of the infusion. On the other hand, the C_max_ of the 24‐h intravenous infusion regimen refers to the concentration reached at 0.5 h.The area under the concentration–time curve within the initial 48 h (AUC_0‐48h_) was estimated for patients administered HD‐MTX, as shown in the equation below:

(7) $${\mathrm{AUC}}_{0-48h}=\frac{\text{Dose}\left(\text{μmol}\right)}{\mathrm{CL}\left(L/h\right)}$$

After the administration of HD‐MTX, the pediatric patients were divided into the nephrotoxicity group and the non‐nephrotoxicity group. The acute kidney injury (AKI) definition from the Kidney Disease: Improving Global Outcomes (KDIGO) group was used to evaluate nephrotoxicity: (1) an increase in the SCr level exceeds 0.3 mg/dL (≥ 26.5 μmol/L) within 48 h, (2) a SCr level that is 1.5 times or higher than the baseline value, and (3) a urine output of less than 0.5 mL/(kg·h) for a period of 6 h [33]. We employed a *t*‐test to ascertain the difference between the groups for quantitative data that adhered to a normal distribution. If the differences between the groups were significant, the area under the curve (AUC) values of the receiver operating characteristic (ROC) curves were used to determine the most reliable predictor of nephrotoxicity and to determine the threshold associated with nephrotoxicity.

## 3 Results

### 3.1 Patient Characteristics

A total of 60 pediatric patients (48 males and 12 females) with a median age of 9.25 years (range from 3.3 to 16.9 years) and a median weight of 24.65 kg (range from 13.2 to 99 kg) were enrolled in this study. A total of 280 plasma concentrations (140 for MTX and 140 for 7‐OHMTX were included in the analysis. The main demographics and clinical characteristics of the enrolled patients are summarized in Table 1.

**TABLE 1:** Main demographics and clinical characteristics of enrolled patients.

Variable	Mean ± SD	Median	Range
Age (years)	9.6 ± 3.5	9.3	3.3–16.9
Weight (kg)	34.2 ± 18.0	24.7	12.2–99.0
BMI (kg/m^2^)	16.1 ± 4.1	15	10.2–32.3
BSA (m^2^)	1.1 ± 0.36	1.2	0.5–1.7
SCr (μmol/L)	42.2 ± 22.5	35.5	12.5–126.8
GFR (mL/min·1.73 m^2^)	146.4 ± 56.3	145.5	48.8–312.9
UA (μmol/L)	240.6 ± 93.1	235.6	19.8–743.7
urea (mmol/L)	3.9 ± 1.9	3.6	1.1–11.9
TP (g/L)	63.2 ± 7.5	63.6	40.3–82.7
TBA (μmol/L)	6.3 ± 5.0	4.9	0.77–29.3
TB (μmol/L)	12.3 ± 6.8	10.3	3.4–42.6
γ‐GGT (U/L)	36.3 ± 24.7	28.3	7.6–167.2
ALT (U/L)	46.0 ± 49.5	30.5	5.2–352.1
AST (U/L)	35.1 ± 30.2	27.0	6.6–207.6
ALP (U/L)	127.7 ± 46.7	116.0	58–256

	*N*%	Percentage
Type of disease		
B‐cell lymphoma	47	78.3
T‐cell lymphoma	13	21.7
Gender (*n*)		
Male	48	80.0
Female	12	20.0

Abbreviation: SD, standard deviation.

### 3.2 PopPK Modeling

**FIGURE 1:**
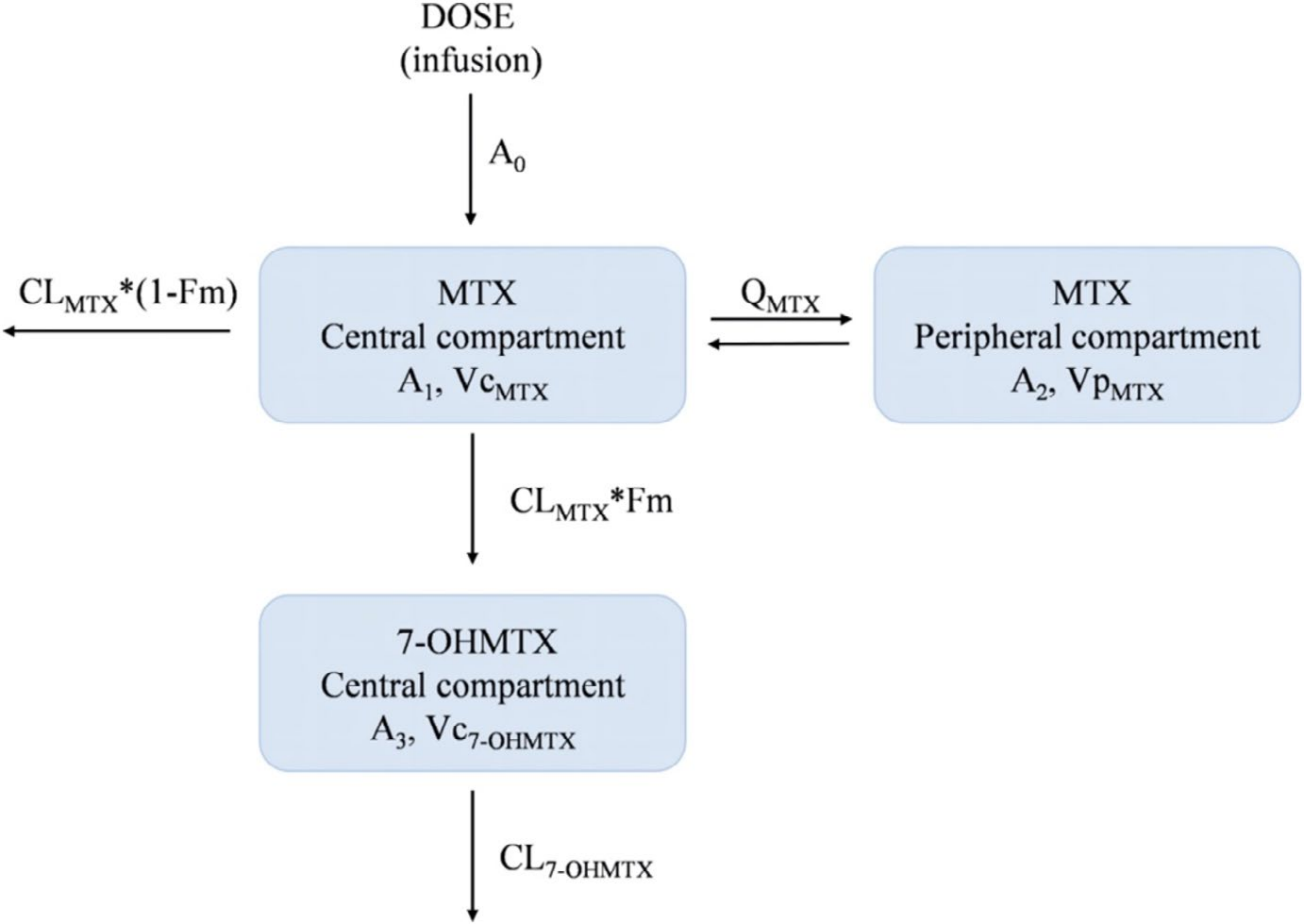
The proposed pharmacokinetic structural model of MTX and 7‐OHMTX in pediatric patients. Dose, dosage in the MTX central compartment (the zero‐order administration rate *A*_0_); A_1_ and A_2_ are the MTX amounts in the central and peripheral compartments, respectively; A_3_ is the 7‐OHMTX amount in the central compartment.

On the basis of the assessment of OFV, AIC, and goodness of fit, it has been shown that a three‐compartment chain model with first‐order absorption and elimination provides the most accurate description of the MTX and 7‐OHMTX data. A schematic representation of the model is shown in Figure 1. The distribution of MTX in the bloodstream was described using a two‐compartment model consisting of compartments 1 and 2. This model contains the central volume of distribution (Vc_MTX_), the peripheral volume of distribution (Vp_MTX_), the central clearance (CL_MTX_), and the intercompartmental clearance (Q_MTX_). The metabolite 7‐OHMTX was formed by the conversion of the parent MTX. Another one‐compartment model (compartments 3) was employed to characterize the disposition of 7‐OHMTX in blood, which included the volume of distribution (Vc_7‐OHMTX_) and the clearance (CL_7‐OHMTX_). It was presumed that 10% of the MTX was eliminated through metabolic conversion to 7‐OHMTX, and the fraction (Fm) of MTX metabolized to 7‐OHMTX was fixed at 0.1.

Proportional residual models could account for the intraindividual variation of both MTX and 7‐OHMTX. Following forward inclusion and backward exclusion criteria for covariate screening, the confirmed covariate AST was finally included into the final PopPK model. The OFV of the final PopPK model was −107.8, which was 10.3 less than that of the base model. Notably, the inclusion of AST as a covariate of Vp_MTX_ resulted in a decrease in interindividual variance from 19.4% to 13.1%, suggesting that 32.5% of BSV in Vp_MTX_ is explained by AST. The final expression is as follows:

$${\mathrm{Vp}}_{\mathrm{MTX}}\left(L\right)=321.6\times {\left(\frac{\mathrm{AST}}{27}\right)}^{\theta_{\mathrm{AST}}}\times e^{\eta^{{\mathrm{Vp}}_{\mathrm{MTX}}}}$$

where *θ*_AST_ represents the effects of AST on Vp_MTX_, $\eta^{{\mathrm{Vp}}_{\mathrm{MTX}}}$ is an independent variable with a mean of 0 and a variance of 0.13. The PopPK parameter estimates for the base and final models and their relative standard errors, covariate effects, and bootstrap results are all summarized in Table 2. The parameter variances and covariate effects of the final model were satisfactory, which suggested that the model estimated the parameters well.

**TABLE 2:** Population pharmacokinetic parameter estimates for base model, final model, and bootstrap results.

Parameter	Base model (RSE%)	Final model (RSE%)	Bootstrap (95% Cl)
*Fixed effects*			
tvVc_MTX_ (L)	261.8 (73.6)	258.2 (71.4)	230.0 (25.5–680.4)
tvVp_MTX_ (L)	370.4 (18.3)	321.6 (20.1)	292.8 (124.1–562.8)
tvCL_MTX_ (L/h)	57.9 (12.4)	56.7 (12.8)	50.4 (34.7–68.0)
tvQ_MTX_ (L/h)	19.6 (23.8)	19.2 (24.2)	14.0 (6.6–23.7)
tvVc_7‐OHMTX_ (L)	182.5 (21.4)	173.4 (21.2)	173.0 (56.2–289.0)
tvCL_7‐OHMTX_ (L/h)	10.8 (13.0)	10.6 (12.6)	10.1 (6.1–12.9)
*θ*_AST_		−0.4 (−47.5)	−0.4 (−0.8–0.7)
*Random effects*			
$\eta^{{\mathrm{Vc}}_{\mathrm{MTX}}}$, %CV	179.5 (112.8)	191.9 (106.6)	273.0 (148.4–699.4)
$\eta^{{\mathrm{Vp}}_{\mathrm{MTX}}}$, %CV	17.0 (51.8)	13.1 (81.2)	14.5 (−8.5–38.5)
$\eta^{{\mathrm{CL}}_{\mathrm{MTX}}}$, %CV	24.0 (30.0)	21.3 (27.2)	19.5 (3.3–34.7)
$\eta^{V_{7-\text{OHMTX}}}$, %CV	50.7 (37.5)	56.1 (42.9)	55.2 (−33.2–143.2)
$\eta^{{\mathrm{CL}}_{7-\text{OHMTX}}}$, %CV	30.3 (19.3)	28.8 (19.5)	25.8 (6.4–45.6)
*σ* MTX	0.26 (10.5)	0.27 (10.8)	0.27 (0.17–0.36)
σ7‐OHMTX	0.25 (14.7)	0.25 (17.1)	0.24 (0.20–0.30)

Abbreviations: CI, confidence interval; RSE%, percentage of relative standard error; tv, population typical value of the parameter; *η*, interindividual variability of parameters; θ_AST_, effect of AST on Vp_MTX_; *σ*, residual variability.

### 3.3 Model Evaluation

**FIGURE 2:**
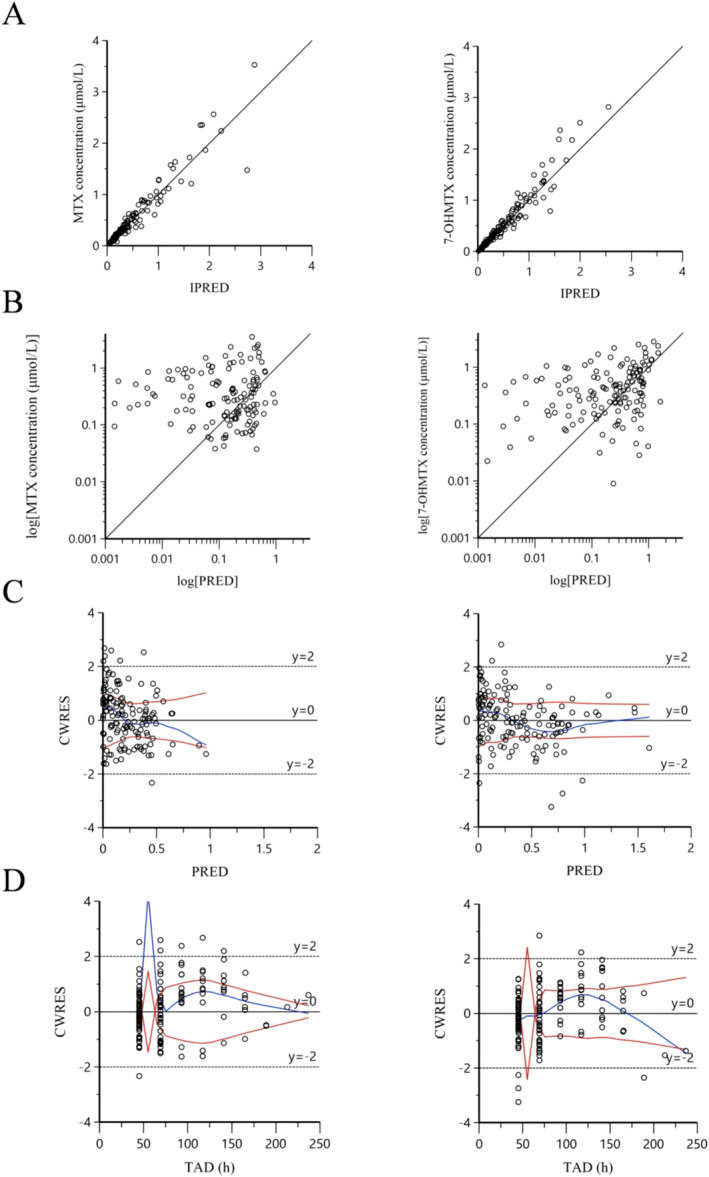
Diagnostic goodness‐of‐fit plots of the final model. The left side displays the diagnostic map of MTX, whereas the right side displays the diagnostic map of 7‐OHMTX. (A) Concentration observations versus individual predictions (IPRED). (B) Concentration observations versus population predictions (PRED); the black solid lines in (A) and (B) mean the line of unity *y* = *x*. (C) Conditional weighted residuals (CWRES) versus time after dose (TAD). (D) CWRES versus PRED; the blue solid lines in (C) and (D) are the trend lines and the red solid lines are the absolute value distribution of the data.

Apart from Vc_MTX_ and *θ*_AST_ values, the final model exhibited low precision with a coefficient of variation ranging from 10% to 20%, which proved that the parameters of the final model were accurate. The GOF plots for both MTX and 7‐OHMTX observations of the final model are shown in Figure 2. Figure 2A,B display scatter plots comparing observation (MTX and 7‐OHMTX concentrations) with population or individual predictions and the solid lines represent unity. The data points were evenly distributed around the line of identity. In Figure 2C,D, plots for CWRES (MTX and 7‐OHMTX) versus time after dose or population predictions are displayed. These plots were utilized to identify any misspecifications that may have been present in the model. The CWRES of the final pharmacokinetic model exhibited a distribution centered around the *y* = 0 line, suggesting the absence of any noticeable systematic bias. The diagnostic plots indicated a good fit for the developed final model.

**FIGURE 3:**
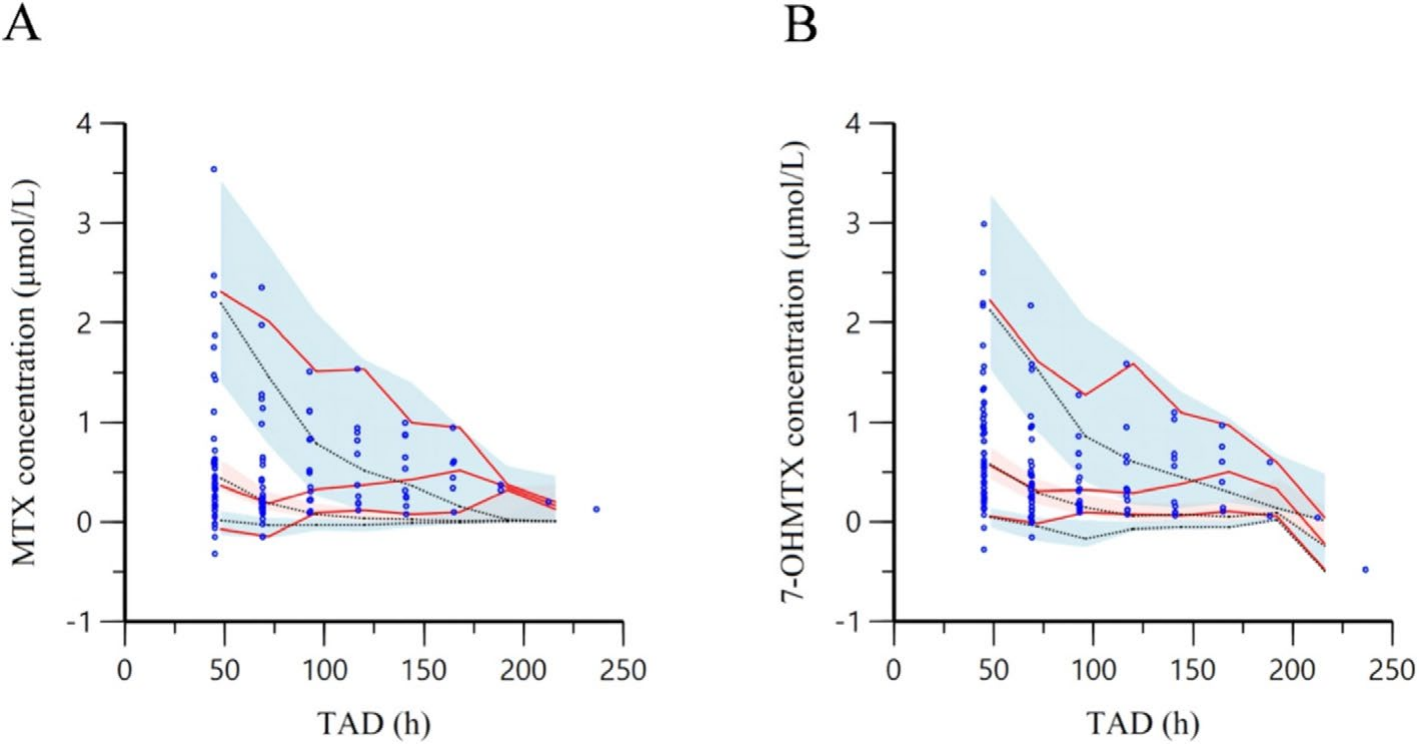
Prediction‐corrected visual prediction checks for the final model of (A) MTX and (B) 7‐OHMTX. Blue dots represent observed concentrations; red lines represent the 2.5th, 50th, and 97.5th quartiles of observed concentrations; black lines represent the 2.5th, 50th, and 97.5th quartiles of simulated values; and shaded areas represent 95% confidence intervals for the 2.5th, 50th, and 97.5th quartiles of simulated values.

The evaluation of 1000 bootstrap runs with a success rate of 99.2% (992 out of 1000 resampling datasets were successfully optimized) revealed acceptable stability of the final PopPK model. Bootstrap results of the median, as well as the 2.5th and 97.5th quantiles of each parameter values, are listed in Table 2. The median values correspond to the final parameter estimations, and the 95% confidence intervals are close to the values acquired during the final data fitting. The pcVPC results are shown in Figure 3. In the VPC plot, it is clear that the observed and fitted values of concentrations of two compunds follow the same trend, which indicates an adequate predictive ability of the final population model.

### 3.4 ROC Curve for Nephrotoxicity

**FIGURE 4:**
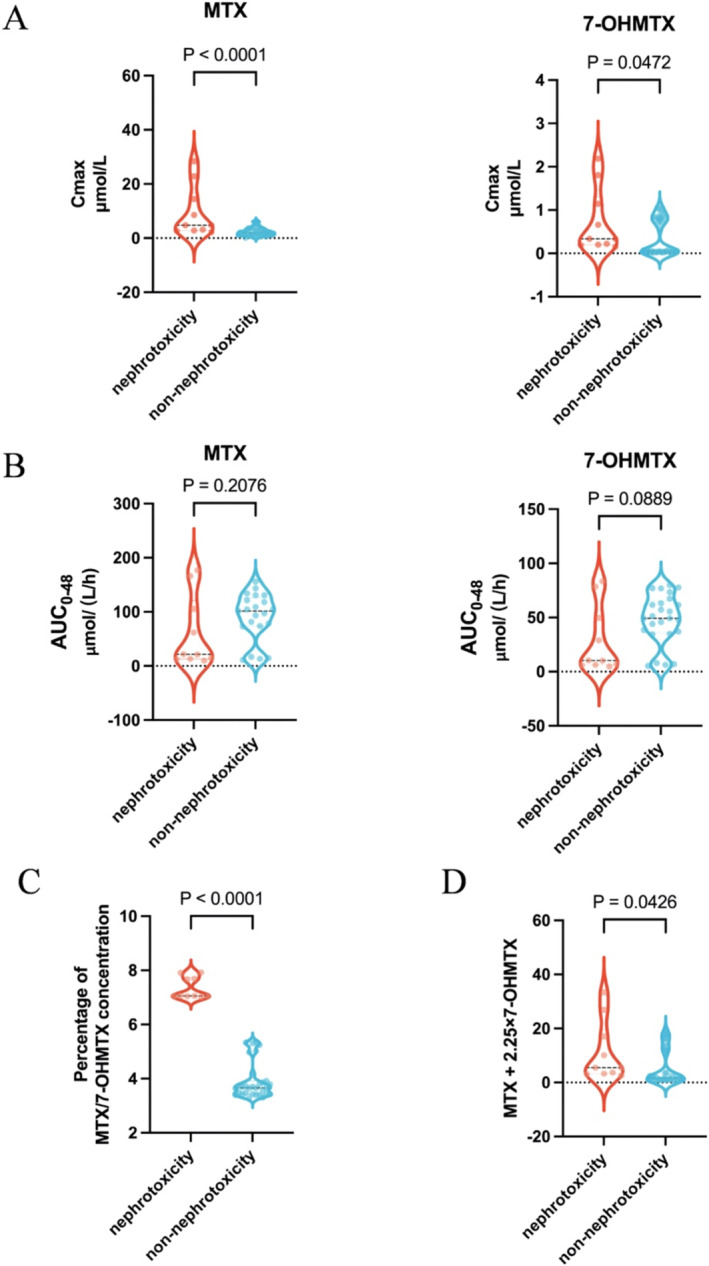
Comparison of (A) C_max,_ (B) AUC_0‐48_ of MTX and 7‐OHMTX, (C) percentage of 7‐‐OHMTX to MTX and (D) MTX + 2.25‐fold 7‐OHMTX concentration between the groups. The median (black line in the violin) values are displayed. Each dot represents a single sample.

**FIGURE 5:**
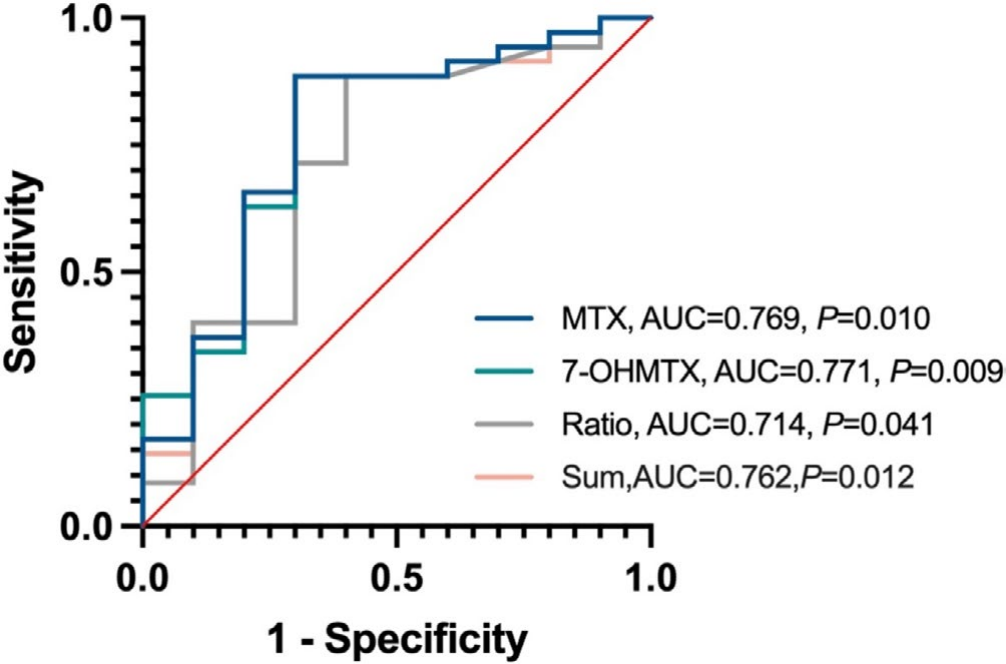
Receiver operating characteristic (ROC) curve for nephrotoxicity.

Patients who did not have pre‐ or posttreatment renal function data available during HD‐MTX chemotherapy were excluded from the subsequent study. A total of 45 patients were systematically monitored for renal function, of which 10 patients suffered from AKI after administration of HD‐MTX. In these 45 patients, Bayesian feedback was employed to forecast *C*_max_ and AUC_0‐48h_. Figure 4 shows that *C*_max_ of MTX and 7‐OHMTX, expressed as the median with interquartile range (IQR), was considerably higher in the group experiencing nephrotoxicity (MTX: 4.76 (2.81–16.54) μmol/L; 7‐OHMTX: 0.33 [0.20–1.31] μmol/L) than the group without nephrotoxicity (MTX:1.99 [1.19–3.15] μmol/L, *p* < 0.0001; 7‐OHMTX: 0.06 (0.03–0.81) μmol/L, *p* = 0.0472). However, there was no significant difference in AUC_0‐48h_ between the nephrotoxicity group (MTX: 21.50 [13.00–120.81] μmol/(L/h); 7‐OHMTX: 10.21 [6.17–56.83] μmol/(L/h)) and the non‐nephrotoxicity group [MTX: 97.01 (16.81–121.41) μmol/(L/h), *p* = 0.2076; 7‐OHMTX: 49.16 (34.40–63.02) μmol/(L/h), *p* = 0.0889] for both MTX and 7‐OHMTX. In addition, we also compared the ratio of 7‐OHMTX to MTX peak concentration between the two groups and the sum of MTX + 2.25‐fold 7‐OHMTX concentration calculated from the difference in LD50 of the two drugs in vitro [19]. The ratio in the nephrotoxic group was significantly larger than that in the non‐nephrotoxic group [7.06 (7.04–7.75) vs. 3.66 (3.43–3.91)], as was the sum of MTX + 2.25 times the 7‐OHMTX concentration in the nephrotoxic group [5.50 (3.17–19.49) vs. 20.86 (14.72–28.65) μmol/L]. Finally, the AUC values for *C*_max_ of MTX and 7‐OHMTX were 0.769 and 0.771, respectively, whereas the AUC values for the ratio of 7‐OHMTX to MTX concentration and the sum of MTX + 2.25 times of 7‐OHMTX concentration were lower, which were 0.714 and 0.763, respectively (Figure 5).

Therefore, the *C*_max_ of MTX and 7‐OHMTX is the reliable predictor for predicting HDMTX nephrotoxicity. The *C*_max_ of MTX and 7‐OHMTX was utilized to forecast the onset of nephrotoxicity following HDMTX treatment using a ROC curve. The minimum threshold for predicting renal toxicity by *C*_max_ of MTX was determined to be 9.26 μmol/L and the minimal threshold of *C*_max_ for 7‐OHMTX was found to be 0.66 μmol/L.

## 4 Discussion

HD‐MTX is effective as part of NHL treatment currently [34], but its main metabolite 7‐OHMTX is highly correlated with toxicity [35], especially renal toxicity. The major reason for this is the poor aqueous solubility of MTX and 7‐OHMTX, particularly at acidic pH, and both compounds may promote the formation of renal lithiasis causing direct kidney injury [36]. In addition, excessive accumulation of the two compounds in the kidney will eventually lead to inflammatory response, oxidative stress, and apoptosis through various pathways, thereby causing renal cell toxicity [19, 20, 21, 22]. Currently, MTX nephrotoxicity is well prevented by clinical protocols for alkalinization and hydration of urine. However, due to the large interindividual variances in the pharmacokinetic course of HD‐MTX, some patients may also have severe adverse reactions after supportive treatment. Although the pharmacokinetics of HD‐MTX has been thoroughly examined in adults and children with acute lymphoblastic leukemia, its pharmacokinetics in children with NHL is not well understood, and the investigation of its metabolite 7‐OHMTX is frequently ignored. At present, there is no clear marker that can predict nephrotoxicity, and the cut‐off value of nephrotoxicity after HD‐MTX treatment has not been reported. Therefore, this research aimed to investigate the correlation between MTX and 7‐OHMTX exposure and safety in pediatric patients with NHL, as well as to identify the most sensitive marker of nephrotoxicity.

The current work employed PopPK approaches to investigate the pharmacokinetic properties of MTX and 7‐OHMTX in pediatric NHL. A joint PopPK model was developed, which accurately fitted the concentration data. This work contributes to the further exploration of the influence of pharmacokinetic features of MTX and 7‐OHMTX, with the goal of providing valuable information for the pediatric clinical application. During the process of developing the base model, the structural models of MTX and 7‐OHMTX were examined using one‐, two‐, and three‐compartment models, respectively. The MTX results were most accurately described by a two‐compartment model with first‐order absorption and elimination. The structural model of 7‐OHMTX was best characterized by a one‐compartment model. In contrast, previous PopPK models established for HD‐MTX in pediatric brain tumors or solid tumors have adequately characterized PK profile of 7‐OHMTX using a two‐compartment model, whereas MTX is described using either a two‐compartment or a three‐compartment model [27, 37]. Each patient received only one dose of MTX, which led to the production of 7‐OHMTX by MTX metabolism. The distribution volume of 7‐OHMTX and the Fm of MTX metabolized to 7‐OHMTX could not be estimated simultaneously. Based on prior research, we set the percentage (Fm) of MTX that was converted to 7‐OHMTX at 10% in the model building [30]. In order to assess the estimation results of various fraction assumptions, we constructed a model based on the fraction fixed 0.25, 0.5, or 1, and the estimation results indicated that the parameters did not vary greatly. In our study, to comprehensively investigate the factors influencing the in vivo behaviors of MTX and 7‐OHMTX, we collected a substantial quantity of information from the study population as covariates. All covariates were introduced into the base model to undergo a stepwise process. After forward inclusion and backward elimination, only AST was identified as the key covariate for Vp_MTX_. Liver function was identified to be a considerable covariate affecting MTX distribution in the pediatric population with NHL, which is consistent with the conclusions of previous PopPK studies [38].

The HDMTX guidelines recommend that intravenous hydration and alkalinization are initiated 12 h or earlier prior to administration [39]; however, adverse events still occur in a small number of patients. In the study on the correlation between HD‐MTX and AKI, it was found that the concentrations of MTX at 48 and 72 h after infusion were correlated with the peak levels of SCr, whereas the concentration at 24 h was not correlated with the degree of AKI [40]. However, in the current study, we focused on nephrotoxicity and discovered that the peak concentrations of MTX and 7‐OHMTX in the group that developed nephrotoxicity were substantially higher than those in the group that did not develop nephrotoxicity, whereas there was no significant difference in AUC_0–48h_. Additionally, we compared the ratio of 7‐OHMTX to MTX peak concentration and the combined concentration of MTX plus 2.25‐fold 7‐OHMTX between the two groups, both of which were considerably increased in the nephrotoxic group. Our study found that the early MTX concentration, specifically the *C*_max_ mentioned in this study, is correlated with nephrotoxicity. Moreover, a previous study in pediatric and adult oncology patients showed that AUC of MTX was associated with an increased risk of nephrotoxicity in adults, while in children, it was associated with hepatotoxicity and neurotoxicity but not nephrotoxicity [41], which is consistent with our finding. However, they did not compare the relationship between *C*_max_, metabolite exposure, and toxicity. Our study provides a more comprehensive comparison of MTX exposure and toxicity.We then plotted the ROC curve for the above four indicators. Among these, the *C*_max_ of MTX and 7‐OHMTX had the greatest AUC values from the ROC curves, suggesting that they are the most sensitive indicators for predicting nephrotoxicity. The maximum limits for preventing nephrotoxicity were determined to be *C*_max_ of 9.26 μmol/L for MTX and *C*_max_ of 0.66 μmol/L for 7‐OHMTX, as concentrations beyond these values are strongly related with an elevated risk of toxicity.

The nephrotoxicity of MTX has become a widespread clinical concern from the perspective of a clinician or pharmacist. Herein, we discuss recommendations regarding the clinical management of the nephrotoxicity of MTX. Prior to initiating MTX treatment, it is important to assess the baseline renal function of patients. This element must be considered while setting the starting dosage. Second, monitoring of *C*_max_ following MTX infusion—at 0.5 h postinfusion for the 24‐h infusion regimen or at the end of infusion for the 3–4 h rapid infusion regimen—may be beneficial in identifying the risk of nephrotoxicity at an earlier stage. This will allow for the adjustment of the initial leucovorin dose or the reinforcement of other supportive measures.

Several limitations should be considered for our study. First, the study had a limited sample size, which was obtained through sparse sampling, and the sample points at the end of infusion were not collected. Therefore, models need to be optimized by larger populations as well as more data at sampling time points in the future research. Second, the absence of long‐term efficacy follow‐up rendered it impossible to evaluate the exposure–response relationship. Specifically, efficacy is a more intricate and multifaceted process than toxicity events. Future studies are necessary to investigate the relationship between exposure and response.

## 5 Conclusion

In conclusion, the joint PopPK model for MTX and its metabolite 7‐OHMTX in pediatric patients with NHL was successfully developed. At the same time, we found that the peak concentrations of MTX and 7‐OHMTX were associated with nephrotoxicity and served as the most sensitive predictors of HD‐MTX‐induced nephrotoxicity. We also proposed the *C*_max_ threshold of 9.26 μmol/L for MTX and 0.66 μmol/L for 7‐OHMTX as the high‐risk critical points for nephrotoxicity. Altogether, this study may contribute crucial insights for ensuring the safe administration of drugs in pediatric clinical practice.

## Author Contributions

**Hao Bing:** software (equal), writing – original draft (lead). **Yi Ma:** data curation (lead), resources (equal). **Jiamin Xu:** validation (equal), visualization (equal). **Qixian Ling:** software (equal), validation (equal). **Yanlong Duan:** supervision (equal), writing – review and editing (equal). **Libo Zhao:** funding acquisition (lead), supervision (equal), writing – review and editing (equal).

## Ethics Statement

This study was in compliance with the Declaration of Helsinki and approved by the Beijing Children's Hospital Medical Science Research Ethics Committee (No. [2022]‐E‐118‐Y).

## Consent

Informed consent was obtained from all subjects involved in the study.

## Conflicts of Interest

The authors declare no conflicts of interest.

## Data Availability

The data presented in this study are available on request from the corresponding author. The data are not publicly available due to ethical reasons as per local guidelines.
